# Using machine learning for the early prediction of sepsis-associated ARDS in the ICU and identification of clinical phenotypes with differential responses to treatment

**DOI:** 10.3389/fphys.2022.1050849

**Published:** 2022-12-12

**Authors:** Yu Bai, Jingen Xia, Xu Huang, Shengsong Chen, Qingyuan Zhan

**Affiliations:** ^1^ Graduate School, Peking Union Medical College, Chinese Academy of Medical Sciences, Beijing, China; ^2^ Department of Pulmonary and Critical Care Medicine, Center of Respiratory Medicine, National Center for Respiratory Medicine, China-Japan Friendship Hospital, Beijing, China

**Keywords:** acute respiratory distress syndrome, sepsis, phenotype, cluster analysis, machine learning

## Abstract

**Background:** An early diagnosis model with clinical phenotype classification is key for the early identification and precise treatment of sepsis-associated acute respiratory distress syndrome (ARDS). This study aimed to: 1) build a machine learning diagnostic model for patients with sepsis-associated ARDS using easily accessible early clinical indicators, 2) conduct rapid classification of clinical phenotypes in this population, and 3) explore the differences in clinical characteristics, outcomes, and treatment responses of different phenotypes.

**Methods:** This study is based on data from the Telehealth Intensive Care Unit (eICU) and Medical Information Mart for Intensive Care IV (MIMIC-IV). We trained and tested the early diagnostic model of sepsis-associated ARDS patients in the eICU. We used key predictive indicators to cluster sepsis-associated ARDS patients and determine the characteristics and clinical outcomes of different phenotypes, as well to explore the differences of in-hospital mortality among different the positive end-expiratory pressure (PEEP) levels in different phenotypes. These results are verified in MIMIC-IV to evaluate whether they are repeatable.

**Results:** Among the diagnostic models constructed in 19,249 sepsis patients and 5,947 sepsis-associated ARDS patients, the AdaBoost (Decision Tree) model achieved the best performance with an area under the receiver operating characteristic curve (AUC) of 0.895, which is higher than that of the traditional Logistic Regression model (Z = −2.40,*p* = 0.013), and the accuracy of 70.06%, sensitivity of 78.11% and specificity of 78.74%. We simultaneously identified three sepsis-associated ARDS phenotypes. Cluster 0 (*n* = 3,669) had the lowest in-hospital mortality rate (6.51%) and fewer laboratory abnormalities (lower WBC (median:15.000 K/mcL), lower blood glucose (median:158.000 mg/dl), lower creatinine (median:1.200 mg/dl), lower lactic acid (median:3.000 mmol/L); *p* < 0.001). Cluster 1 (*n* = 1,554) had the highest in-hospital mortality rate (75.29%) and the most laboratory abnormalities (higher WBC (median:18.300 K/mcL), higher blood glucose (median:188.000 mg/dl), higher creatinine (median:2.300 mg/dl), higher lactic acid (median:3.900 mmol/L); *p* < 0.001). Cluster 2 (*n* = 724) had the most complex condition, with a moderate in-hospital mortality rate (29.7%) and the longest intensive care unit stay. In Clusters 0 and 1, patients with high PEEP had higher in-hospital mortality rate than those with low PEEP, but the opposite trend was seen in Cluster 2. These results were repeatable in 11,935 patients with sepsis and 2,699 patients with sepsis-associated ARDS patients in the MIMIC-IV cohort.

**Conclusion:** A machine learning diagnostic model of sepsis-associated ARDS patients was established. Three phenotypes with different clinical features and outcomes were clustered, and these had different therapeutic responses. These findings are helpful for the early and rapid identification of sepsis-associated ARDS patients and their precise individualized treatment.

## Background

Acute respiratory distress syndrome (ARDS), one of the most common respiratory syndromes in intensive care unit (ICU), is characterized by rapidly progressive respiratory failure, pulmonary edema, diffuse alveolar damage, and inflammatory cell infiltration (Thompson et al., 2017). Although many basic and clinical studies in the past 50 years have continuously clarified the pathophysiological mechanisms of ARDS and proposed new treatments (Meyer et al., 2021), the mortality rate of ARDS patients is still as high as 40% (Bellani et al., 2016; Huang et al., 2020), and at least 50% will develop complications of varying degrees (Huang et al., 2020). Sepsis is a common risk factor for ARDS. Studies have shown that sepsis-associated ARDS is more serious than non-sepsis-associated ARDS, because it is more difficult to recover from lung injury caused by the former and its mortality rate is relatively high (Zhao et al., 2020). Improving the diagnosis and phenotypic classification of ARDS, as well as performing precision medicine, remain some of the main research directions in the field of respiratory critical care. In particular, high mortality conditions such as sepsis-associated ARDS with high mortality should be more actively diagnosed to reduce burden. It is important to predict the occurrence of sepsis-related ARDS at an early time, as well as to classify the clinical subgroups in the early stage and improve the effectiveness of interventions for target subgroups.

The use of artificial intelligence in the medical field has recently become more frequent. Using machine learning to build disease prediction models can predict the occurrence of diseases at an early stage. Machine learning can also cluster diseases to aid in making clinical decisions. Artificial intelligence may have a role in guiding clinicians to make important decisions, but there are some challenges as well as obstacles in ICU, such as the weak interpretability of the model, lack of robustness, and ethical concerns (Yoon et al., 2022). At present, the diagnosis and prediction of sepsis-associated ARDS by machine learning has not been studied, and there are no specific studies on clinical subtypes of sepsis-associated ARDS, with most subtype studies focusing on the entire ARDS population. Seymour et al. (2019) identified four clinical phenotypes of sepsis through cluster analysis, which were related to host response patterns and clinical outcomes. Calfee et al. (2014) classified ARDS patients into classic hyperinflammatory and hypoinflammatory types, but the definition of this biological phenotype requires the use of plasma biomarkers as class-defining variables, such as sTNFR-1 and interleukin (ILs), which are not routinely available and cannot be quickly quantified at bedside. Therefore, the clinical applicability of this classification system may be limited. In 2021, Liu et al. (2021) proposed a new division of ARDS patients into three clinical phenotypes using rapidly accessible clinical indicators. They concluded that the clinical phenotypes of ARDS were associated with different treatment responses to randomized interventions, but this study was based on the entire ARDS population. Studies in the past few years have used clustering methods to analyze the entire subtype of ARDS or sepsis but have not identified the clinical subtype of sepsis-associated ARDS. This classification is more detailed and precise, and it can easily be applied for clinical use.

In this study, we used rapid and easy-to-obtain early clinical indicators in a large-scale population of sepsis and sepsis-associated ARDS patients to build an early diagnostic model for these patients. A rapid early clinical subgroup classification was adopted for this population, assuming that these phenotypes have different clinical outcomes and different treatment responses. We validated this model in another large sepsis and sepsis-associated ARDS population.

## Methods

### Study design

**FIGURE 1:**
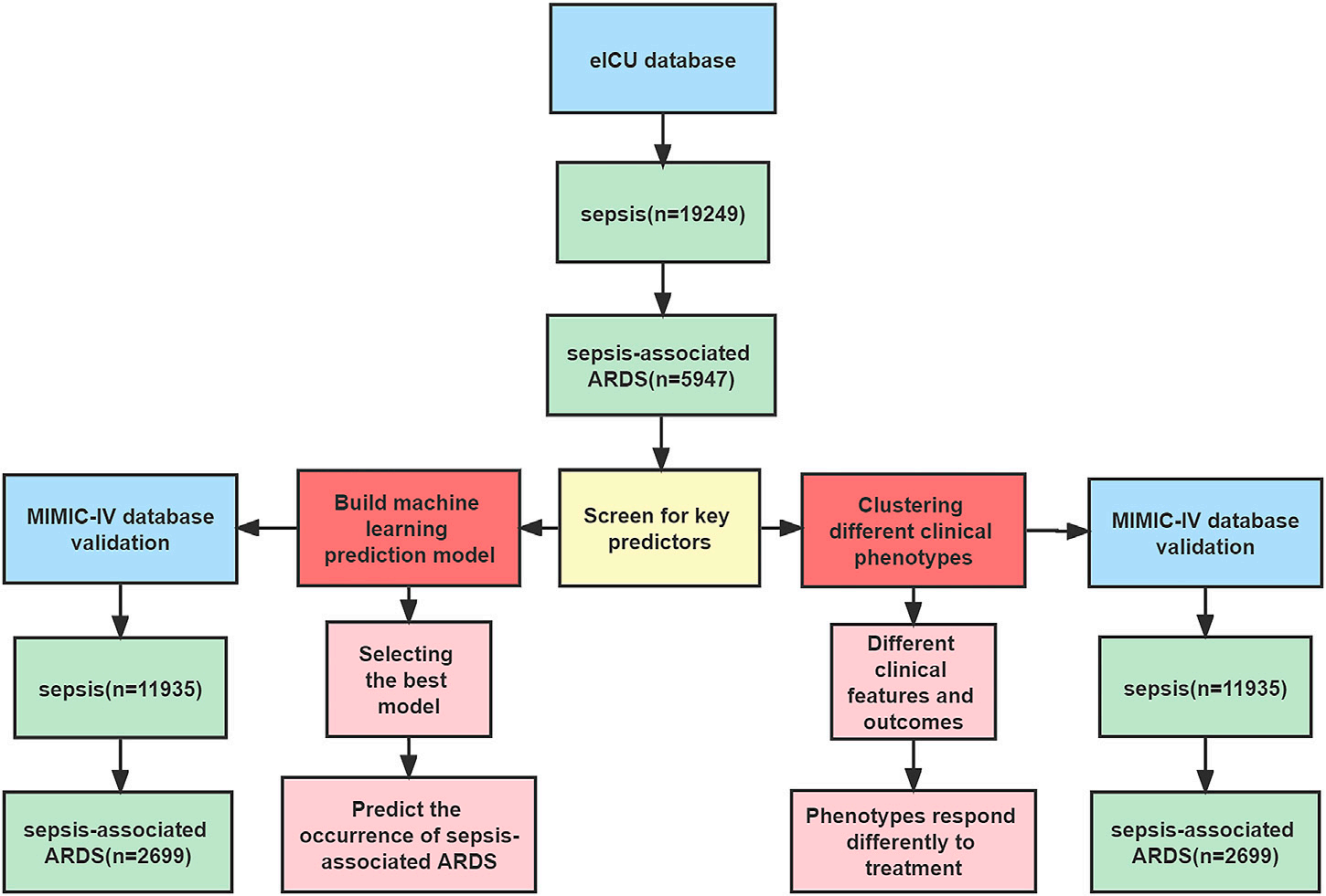
Flowchart of this study.

Our study included two large databases and was a retrospective study. First, we trained an early diagnosis model for patients with sepsis-associated ARDS in the Telehealth Intensive Care Unit (eICU) and tested the stability of the model. At the same time, subgroup clustering of sepsis-associated ARDS patients was performed using key predictors (variables that are ultimately included in the models) to determine differences in characteristics and clinical outcomes among different phenotypes, as well as to explore the differences in in-hospital mortality between different phenotypes with different early positive end-expiratory pressure (PEEP) levels. These results were validated in Medical Information Mart for Intensive Care IV (MIMIC-IV) to assess whether they were reproducible. The specific process is shown in Figure 1.

### Patient population

Our training and testing data is based on the eICU database, a multicenter ICU database with over 200,000 electronic medical records from 335 units at 208 hospitals across the United States between 2014 and 2015 (Pollard et al., 2018). We used the International Classification of Diseases, Ninth Revision, Clinical Modification (ICD-9-CM) Codes to retrieve sepsis and ARDS patients, and sepsis-associated ARDS patients were obtained by associating them with “patientunitstayid.” The external validation dataset is based on the MIMIC-IV database, which is a large, single-center, open-access database of 76,540 ICU admissions between 2008 and 2019 (Johnson et al., 2021). We used the International Classification of Diseases, Ninth and 10th Revision, Clinical Modification (ICD-9-CM,I CD-10-CM) Codes to retrieve sepsis patients and ARDS patients, and sepsis-associated ARDS patients were obtained by correlation with “stay_id.” We extracted data from the medical ICUs (MICU), surgical ICUs (SICU), and medical-surgical ICUs (Med-Surg ICU) in the two databases. Specialist critical care units such as cardiothoracic and cardio-surgical ICUs were excluded because of their specific patient cohorts with distinct presentations of sepsis. In the MIMIC-IV, sepsis was diagnosed based on the Sepsis-3 criteria, including suspected infection and a SOFA score≥2 (Seymour et al., 2016). In the eICU, sepsis and ARDS was identified according to the admission diagnosis recorded on the Acute Physiology and Chronic Health Evaluation IV (APACHE IV) dataset. We excluded patients who were admitted for less than 24 h or diagnosed with ARDS within 24 h after admission. Patient identities in MIMIC-IV database include subject_id, hadm_id and stay_id. Subject_id is unique to a patient, even if the patient has been admitted multiple times. Hadm_id is unique to a patient hospital stay, each hospital stay has a different hadm_id. Stay_id is unique to a patient ward stay, each ICU stay has a different stay_id. In MIMIC-IV database, we correlated subject_id and stay_id, the subject_id is selected only once, and excluded multiple analyses of the same patient. In eICU, only one of the methods for identifying patients is patientunitstayid, similar to the subject_id in MIMIC database. In the eICU database, we excluded multiple analyses of the same patient by screening out patients corresponding to repeated patientuistayid.

We accessed the eICU and MIMIC-IV after completion of the Protecting Human Research Participants exam (Record ID: 44151052). This study was conducted in accordance with the principles of the Declaration of Helsinki in 2013, and patients provided consent to have their data captured in the two databases. Thus, the ethical approval statement was waived in this study, as the data in the eICU and MIMIC-IV database were unidentifiable.

### Predictor variables

We used structured query language programming in Navicat Premium (version 15) to extract clinical data from the eICU and MIMIC-IV database. We collected information related to the patients’ demographic characteristics, history of diseases, laboratory findings, severity scores of illness and outcomes. After evaluating data availability and clinical variable missing rates in both databases, 27 variables were selected as candidate training variables: age, gender, body mass index (BMI), hypertension, diabetes, cancer, acute physiology and chronic health evaluation IV (APACHE IV)/acute physiology score III (APSIII), and the maximum and minimum values of albumin, bicarbnonate (HCO_3_), lactate, bilirubin, creatinine, glucose, platelet, prothrombin time (PT), blood urea nitrogen (BUN), and white blood cell count (WBC). In order to reflect the early stage and timeliness of the model, we extracted the maximum and minimum values of laboratory indicators within 24 h after admission. Since APACHE IV index was not found in MIMIC-IV database, APSIII was used instead.

### Statistical analysis

We cleaned the data and interpolated the missing values using the multiple imputation method, which is recognized as a better approach to deal with missing observations in both outcome and independent variables to ensure data integrity prior to analysis. For single variables, the specific methods of interpolation include linear regression, logistic regression and predicted mean method (pmm). By default, five data sets were generated. We averaged the data of these five data sets. We also standardized data prior to cluster analysis. The normal distribution of continuous variables was determined using the Kolmogorov-Smirnov test. The skewed distributed variables were expressed as the median and interquartile range (IQR), while categorical variables were expressed as number and percentages. Continuous variables between groups were compared by Mann-Whitney test or the Kruskal–Wallis test, as appropriate. Categorical variables between groups were compared by Pearson’s chi-squared test or Fisher’s exact test, as appropriate. SPSS23.0 software was used for statistical analysis.

### Model training and testing

In building a sepsis-associated ARDS diagnostic model, we divided the patients in the eICU at a 7:3 ratio into a training set and a test set, respectively. Weight by correlation algorithm was used to screen the key predictors of sepsis-associated ARDS. We have trained five models: Naive Bayes, Logistic Regression, Gradient Boosted Trees, AdaBoost (Decision Tree), and Random Forest. Model performance was measured using the area under the receiver operating characteristic curve (AUROC), accuracy, sensitivity and specificity. We used the Youden index maximum method to determine the optimal working point of the ROC curve and the corresponding sensitivity and specificity. Area under the receiver operating characteristic curves (AUCs) were compared using DeLong’s method. After the best performance model is selected, external verification is carried out in MIMIC-IV. In the construction of sepsis-associated ARDS clinical subgroup classification, we used K-means clustering model through key predictors. After the optimal phenotypes were derived, the results were visualized using a scatter plot and centroid chart. The clinical characteristics and outcomes of different phenotypes were analyzed, and the differences of in-hospital mortality of different PEEP levels in different phenotypes were compared. The same method was used for external validation in MIMIC-IV. The overall model was built using RapidMiner Studio 9.10.001 and python 3.7.

## Results

### Participants

A total of 19,249 patients with sepsis were included in the eICU database, including 5,947 patients with sepsis-associated ARDS (Table 1). Meanwhile, 11,935 patients with sepsis were included in the MIMIC-IV database, including 2,699 patients with sepsis-associated ARDS (Table 2). Source of infection in septic patients and in septic-associated ARDS from gastrointestinal, cutaneous/soft tissue, pulmonary, gynecologic, renal, urinary tract infection, abdominal infection and unknown. In the eICU database, the overall population of sepsis patients had an in-hospital mortality rate of 16.84% and median ICU stay of 2.3 days, whereas for patients with sepsis-associated ARDS, these values were 27.31% and 3.4 days, respectively. For the MIMIC-IV database, the overall population of sepsis patients had an in-hospital mortality rate of 16.51% and median ICU stay of 2.1 days, whereas for patients with sepsis-associated ARDS, these values were 22.05% and 2.9 days, respectively.

**TABLE 1:** Baseline demographic and clinical characteristics of patients from the eICU database.

	Total sepsis (n = 19,249)	No sepsis-associated ARDS (*n* = 13,302)	Sepsis-associated ARDS (*n* = 5,947)	*p*
Demographics				
Age, year	67.000 (55.0.78.0)	67.000 (55.0.78.0)	67.000 (56.0.78.0)	0.045∗
Male, n (%)	9,935 (51.61%)	6,785 (51.01%)	3,150 (52.97%)	0.012∗
BMI, kg/m2	27.359 (23.0.33.0)	27.300 (23.0.32.9)	27.500 (23.1.33.2)	0.191
Comorbidities				
Hypertension, n (%)	10,063 (52.28%)	6,972 (52.41%)	3,091 (51.98%)	0.575
Diabetes, n (%)	3,132 (16.27%)	2,228 (16.75%)	904 (15.20%)	0.007∗∗
Cancer, n (%)	3,592 (18.66%)	2,505 (18.83%)	1,087 (18.28%)	0.362
Myocardial infarction, n (%)	1,571 (8.16%)	1,024 (7.70%)	547 (9.20%)	<0.001∗∗
CHF, n (%)	3,092 (16.06%)	2075 (15.60%)	1,017 (17.10%)	0.009∗∗
COPD, n (%)	2,975 (15.46%)	1982 (14.90%)	993 (16.70%)	<0.001∗∗
Asthma, n (%)	1,418 (7.37%)	931 (7.00%)	487 (8.19%)	0.003∗∗
Systemic use of hormones, n (%)	4,827 (25.08%)	3,318 (24.94%)	1,509 (25.37%)	0.524
Systemic use of immunosuppressants, n (%)	1,168 (6.07%)	705 (5.30%)	463 (7.79%)	<0.001∗∗
Vitals				
Maximum WBC, K/mcL	15.700 (10.8.21.3)	15.600 (10.7.21.3)	15.900 (10.9.21.4)	0.054
Minimum WBC, K/mcL	11.200 (7.3.15.7)	11.100 (7.3.15.7)	11.200 (7.4.15.6)	0.713
Maximum albumin, g/dL	2.966 (2.6.3.4)	3.000 (2.6.3.4)	3.000 (2.5.3.3)	<0.001∗∗
Minimum albumin, g/dL	2.623 (2.2.3.0)	2.600 (2.2.3.0)	2.600 (2.2.2.9)	<0.001∗∗
Maximum HCO_3_, mmol/L	23.297 (22.0.23.3)	23.300 (23.0.23.3)	23.300 (21.0.27.0)	<0.001∗∗
Minimum HCO_3_, mmol/L	20.739 (20.0.21.0)	20.700 (20.7.20.7)	20.700 (18.0.24.0)	<0.001∗∗
Maximum lactate, mmol/L	3.394 (1.8.3.4)	3.200 (1.7.3.4)	3.400 (1.8.3.6)	<0.001∗∗
Minimum lactate, mmol/L	2.000 (1.2.2.0)	1.900 (1.1.2.0)	2.000 (1.2.2.0)	<0.001∗∗
Maximum creatinine, mg/dL	1.460 (0.9.2.4)	1.500 (1.0.2.5)	1.500 (0.9.2.4)	0.024∗
Minimum creatinine, mg/dL	1.100 (0.7.1.8)	1.100 (0.8.1.9)	1.100 (0.8.1.7)	0.009∗∗
Maximum BUN, mg/dL	30.000 (18.0.47.0)	29.000 (18.0.47.0)	31.000 (19.0.47.0)	<0.001∗∗
Minimum BUN, mg/dL	23.000 (14.0.37.0)	23.000 (13.0.37.0)	24.000 (15.0.38.0)	0.004∗∗
Maximum glucose, mg/dL	156.000 (122.0.209.0)	152.000 (120.0.204.0)	164.000 (127.0.219.0)	<0.001∗∗
Minimum glucose, mg/dL	111.000 (91.0.138.0)	110.000 (91.0.135.3)	113.000 (91.0.142.0)	<0.001∗∗
Maximum total bilirubin, mg/dL	0.900 (0.5.1.4)	0.900 (0.5.1.4)	0.900 (0.5.1.4)	0.193
Minimum total bilirubin, mg/dL	0.700 (0.4.1.1)	0.700 (0.4.1.1)	0.700 (0.4.1.1)	0.954
Maximum platelet, K/mcL	225.000 (157.0.302.0)	224.000 (156.0.301.3)	230.000 (162.0.302.0)	0.011∗
Minimum platelet, K/mcL	181.000 (123.0.245.0)	180.000 (123.0.244.0)	184.000 (123.0.245.0)	0.356
Maximum PT, sec	19.600 (14.7.19.6)	19.600 (14.7.19.6)	19.600 (14.5.19.6)	0.621
Minimum PT, sec	17.000 (14.2.17.0)	17.000 (14.3.17.0)	17.000 (14.0.17.0)	0.043∗
APACHE IV	68.006 (51.0.79.0)	67.000 (49.0.75.0)	68.000 (58.0.91.0)	<0.001∗∗
Complications				
AKI, n (%)	6,825 (35.46%)	4,002 (30.09%)	2,823 (47.47%)	<0.001∗∗
Shock, n (%)	2,213 (11.50%)	1,286 (9.67%)	927 (15.49%)	<0.001∗∗
Vasopressor treatment, n (%)	6,559 (34.07%)	4,056 (30.49%)	2,503 (42.09%)	<0.001∗∗
DIC, n (%)	114 (0.59%)	50 (0.38%)	64 (1.08%)	<0.001∗∗
Outcome				
LOS, days	2.300 (1.2.4.6)	2.000 (1.1.3.7)	3.400 (1.7.7.0)	<0.001∗∗
Mortality, n (%)	3,242 (16.84%)	1,618 (12.16%)	1,624 (27.31%)	<0.001∗∗

∗ *p* < 0.05 ∗∗ *p* < 0.01 If the variable is a continuous value, it is expressed as the median (interquartile range), and if the variable is a categorical value, it is expressed as a number (percentage of the total). *p* values represent the comparison between the no sepsis-associated ARDS group and the sepsis-associated ARDS group. BMI: body mass index; WBC: white blood count; HCO_3_: bicarbonate; BUN: blood urea nitrogen; PT: prothrombin time; APACHE IV: acute physiology and chronic health evaluation iv; LOS: length of stay; CHF: congestive heart failure; COPD: chronic obstructive pulmonary disease; AKI: acute kidney injury; DIC: disseminated intravascular coagulation.

**TABLE 2:** Baseline demographic and clinical characteristics of patients from the MIMIC-IV database.

	Total sepsis (*n* = 11,935)	No sepsis-associated ARDS (*n* = 9,236)	Sepsis-associated ARDS (*n* = 2,699)	*p*
Demographics				
Age, years	65.000 (54.0.76.0)	66.000 (54.0.77.0)	63.000 (53.0.75.0)	<0.001∗∗
Male, n (%)	6,533 (54.74%)	5,073 (54.93%)	1,460 (54.09%)	0.445
BMI, kg/m^2^	27.774 (23.3.31.5)	27.600 (23.2.31.5)	28.300 (23.7.31.6)	<0.001∗∗
Comorbidities				
Hypertension, n (%)	6,346 (53.17%)	4,938 (53.47%)	1,408 (52.15%)	0.075
Diabetes, n (%)	3,774 (31.62%)	2,988 (32.35%)	786 (29.12%)	0.002∗∗
Cancer, n (%)	1,262 (10.57%)	974 (10.55%)	287 (10.63%)	0.637
Myocardial infarction, n (%)	1,006 (8.43%)	766 (8.29%)	240 (8.89%)	0.325
CHF, n (%)	2017 (16.90%)	1,496 (16.20%)	521 (19.30%)	<0.001∗∗
COPD, n (%)	2,441 (20.45%)	1856 (20.10%)	585 (21.67%)	0.074
Asthma, n (%)	1,130 (9.47%)	858 (9.29%)	272 (10.08%)	0.219
Systemic use of hormones, n (%)	2,287 (19.16%)	1,551 (16.79%)	736 (27.27%)	<0.001∗∗
Systemic use of immunosuppressants, n (%)	609 (5.10%)	452 (4.89%)	157 (5.82%)	0.055
Vitals				
Maximum WBC, K/mcL	13.100 (8.7.19.0)	13.300 (8.8.19.0)	12.700 (8.5.18.7)	0.038*
Minimum WBC, K/mcL	9.700 (6.4.14.1)	9.800 (6.4.14.2)	9.400 (6.3.13.7)	<0.010∗∗
Maximum albumin, g/dL	3.029 (3.0.3.0)	3.000 (3.0.3.0)	3.000 (3.0.3.1)	0.608
Minimum albumin, g/dL	2.9232.900.2.923]	2.900 (2.9.2.9)	2.900 (2.9.3.0)	0.937
Maximum HCO_3_, mmol/L	24.000 (21.0.27.0)	24.000 (21.0.26.0)	24.000 (21.0.28.0)	<0.001∗∗
Minimum HCO_3_, mmol/L	21.000 (18.0.24.0)	21.000 (18.0.24.0)	21.000 (18.0.25.0)	<0.001∗∗
Maximum lactate, mmol/L	3.238 (1.9.3.2)	3.200 (2.0.3.2)	3.200 (1.7.3.2)	<0.001∗∗
Minimum lactate, mmol/L	1.958 (1.4.2.0)	2.000 (1.5.2.0)	2.000 (1.2.2.0)	<0.001∗∗
Maximum creatinine, mg/dL	1.300 (0.9.2.2)	1.300 (0.9.2.1)	1.400 (0.9.2.5)	<0.001∗∗
Minimum creatinine, mg/dL	1.100 (0.7.1.8)	1.100 (0.7.1.8)	1.100 (0.7.2.0)	0.003∗∗
Maximum BUN, mg/dL	27.000 (17.0.45.0)	26.000 (16.0.44.0)	31.000 (19.0.51.0)	<0.001∗∗
Minimum BUN, mg/dL	22.000 (14.0.38.0)	22.000 (13.0.36.0)	25.000 (15.0.42.0)	<0.001∗∗
Maximum glucose, mg/dL	144.000 (115.0.196.0)	142.000 (114.0.192.0)	150.000 (118.0.206.0)	<0.001∗∗
Minimum glucose, mg/dL	107.000 (89.0.132.0)	107.000 (90.0.131.0)	107.000 (89.0.133.0)	0.882
Maximum total bilirubin, mg/dL	1.800 (0.6.2.4)	1.900 (0.6.2.4)	1.500 (0.5.2.4)	<0.001∗∗
Minimum total bilirubin, mg/dL	1.400 (0.5.2.0)	1.500 (0.5.2.0)	1.200 (0.4.2.0)	<0.001∗∗
Maximum platelet, K/mcL	212.000 (143.0.299.0)	211.000 (143.0.296.0)	216.000 (140.0.307.0)	0.25
Minimum platelet, K/mcL	176.0 (114.0.252.0)	175.000 (114.0.250.0)	178.000 (112.0.258.0)	0.757
Maximum PT, sec	16.100 (13.6.20.1)	16.100 (13.6.20.1)	16.100 (13.4.20.2)	0.763
Minimum PT, sec	14.8 (12.9.16.8)	14.800 (12.9.16.8)	14.800 (12.7.17.4)	0.895
APSIII	51.000 (38.0.68.0)	49.000 (37.0.65.0)	57.000 (42.0.77.0)	<0.001∗∗
Complications				
AKI, n (%)	4,852 (40.65%)	3,481 (37.69%)	1,371 (50.80%)	<0.001∗∗
Shock, n (%)	1,591 (13.33%)	1,062 (11.50%)	529 (19.60%)	<0.001∗∗
Vasopressor treatment, n (%)	4,151 (34.78%)	2,956 (32.01%)	1,195 (44.28%)	<0.001∗∗
DIC, n (%)	207 (1.73%)	129 (1.40%)	78 (2.89%)	<0.001∗∗
Outcome				
LOS, days	2.110 (1.2.4.1)	2.000 (1.1.3.7)	2.900 (1.5.6.7)	<0.001∗∗
Mortality, n (%)	1971 (16.51%)	1,376 (14.90%)	595 (22.05%)	<0.001∗∗

*p < 0.05 ∗∗ *p* < 0.01 If the variable is a continuous value, it is expressed as the median (interquartile range), and if the variable is a categorical value, it is expressed as a number (percentage of the total). *p* values represent the comparison between the no sepsis-associated ARDS group and the sepsis-associated ARDS group.BMI: body mass index; WBC: white blood count; HCO_3_: bicarbonate; BUN: blood urea nitrogen; PT: prothrombin time; APSIII: acute physiology score iii; LOS: length of stay; CHF: congestive heart failure; COPD: chronic obstructive pulmonary disease; AKI: acute kidney injury; DIC: disseminated intravascular coagulation.

### Establishment and verification of sepsis-associated ARDS diagnostic model

**FIGURE 2:**
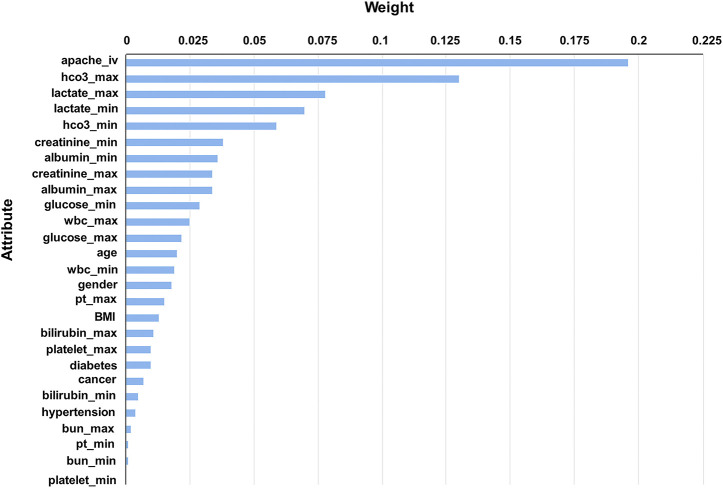
Feature screening of sepsis-associated ARDS diagnostic model.

**FIGURE 3:**
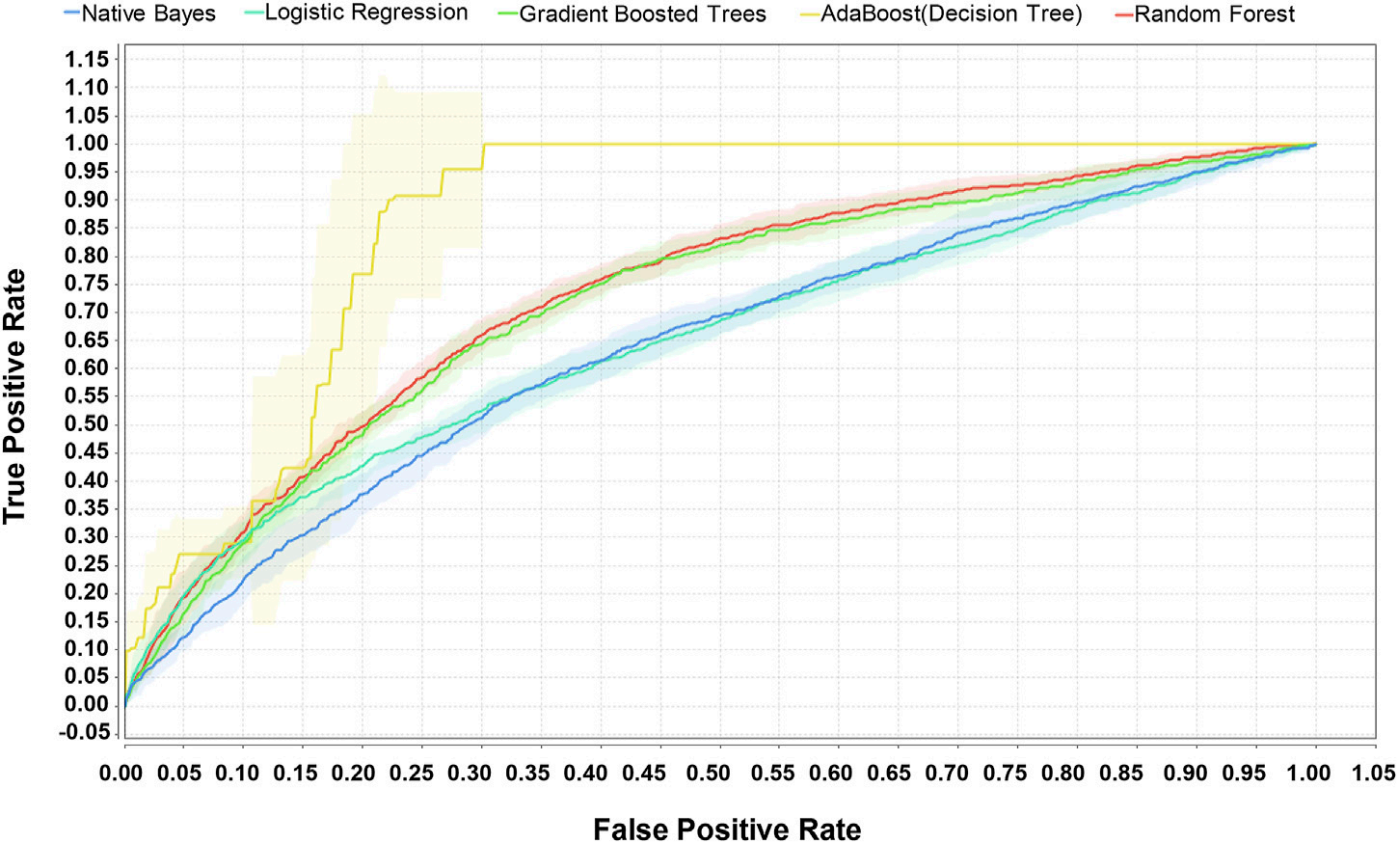
Comparison of ROC of five machine learning methods for predicting sepsis-associated ARDS in eICU test set. The AUC of the five models in the eICU test cohort are as follows: Naive Bayes: 0.644, Logistic Regression: 0.653, Gradient Boosted Trees:0.736, AdaBoost (Decision Tree): 0.895, and Random Forest: 0.763.

**FIGURE 4:**
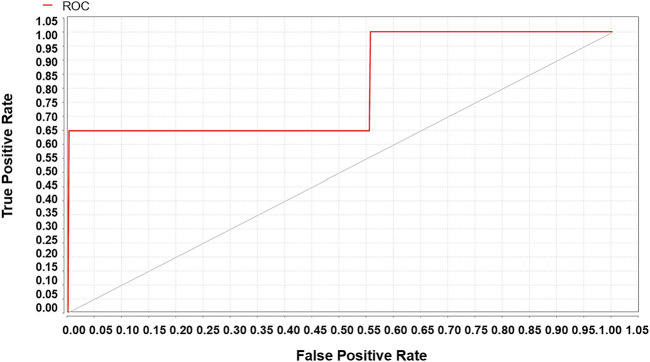
The MIMIC-IV verification set validates the sepsis-associated ARDS ROC of the AdaBoost (Decision Tree) model, AUC = 0.804.

A total of 19,249 sepsis patients, including 5,947 patients with sepsis-associated ARDS, were selected from the eICU database. The entire cohort was randomly divided into a training cohort (13,474, 70%) and a test cohort (5,775, 30%). We used the weight by correlation algorithm to select the key predictors among the 27 input variables. We selected 14 key predictors (Figure 2) with high weight (>0.02), which are APACHE IV and minimum and maximum HCO_3_, lactate, creatinine, albumin, glucose, WBC, and age. Using 14 variables as input factors, five machine learning methods, including Naive Bayes, Logistic Regression, Gradient Boosted Trees, AdaBoost (Decision Tree), and Random Forest were established to predict the occurrence of sepsis-associated ARDS. In the eICU test queue, the AUC and corresponding accuracies, sensitivities and specificities of the five models are as follows: Naive Bayes: 0.644 (95%CI: 0.632–0.656) (68.89% (95%CI:68.69%–69.10%) accuracy, 72.67% (95%CI:72.42%–72.92%) sensitivity, 48.75% (95%CI:45.83%–51.67%) specificity), Logistic Regression: 0.653 (95%CI:0.641–0.667) (71.34% (95%CI:71.20%–71.48%) accuracy, 71.95% (95%CI:70.42%–73.48%) sensitivity, 44.18% (95%CI:43.23%–45.13%) specificity), Gradient Boosted Trees: 0.736 (95%CI:0.697–0.795) (67.19% (95%CI:66.46%–68.00%) accuracy, 71.43% (95%CI:70.12%–72.75%) sensitivity, 65.29% (95%CI:64.10%–66.48%) specificity), AdaBoost (Decision Tree): 0.895 (95%CI:0.834–0.936) (70.06% (95%CI:68.87%–71.28%) accuracy, 78.11% (95%CI:74.23%–81.23%) sensitivity, 78.74% (95%CI:76.13%–81.35%) specificity), and Random Forest: 0.763 (95%CI:0.731–0.820) (69.16% (95%CI:67.11%–71.21%) accuracy, 74.92% (95%CI:70.85%–78.99%) sensitivity, 69.38% (95%CI:66.82%–72.03%) specificity). The performance comparison of the models is shown in Table 3; Figure 3. From the performance index of the models, the AUC value of AdaBoost (DecisionTree) is the highest, which is higher than that of the traditional Logistic Regression model (Z = -2.40,*p* = 0.013), and the sensitivity, specificity and accuracy are higher. We selected AdaBoost (Decision Tree), with the best performance as the final model. We performed external verification in the MIMIC-IV cohort. A total of 11,935 septic patients were selected in the MIMIC-IV database, of which 2,699 were sepsis-associated ARDS patients. Since there is no APACHE IV in MIMIC-IV, we used APSIII instead. In the verification set, 14 key predictors were inputted into the AdaBoost (Decision Tree) model, and the resulting AUC is the 0.804. The ROC curve in MIMIC-IV external verification set is shown in Figure 4.

**TABLE 3:** Performance of the five machine learning models for predicting sepsis-associated ARDS.

Models	AUC (95%CI)	Accuracy (95%CI)	Sensitivity (95%CI)	Specificity (95%CI)
Naive Bayes	0.644 (0.632–0.656)	68.89% (68.69%–69.10%)	72.67% (72.42%–72.92%)	48.75% (45.83%–51.67%)
Logistic Regression	0.653 (0.641–0.667)	71.34% (71.20%–71.48%)	71.95% (70.42%–73.48%)	44.18% (43.23%–45.13%)
Gradient Boosted Trees	0.736 (0.697–0.795)	67.19% (66.46%–68.00%)	71.43% (70.12%–72.75%)	65.29% (64.10%–66.48%)
AdaBoost (Decision Tree)	0.895 (0.834–0.936)	70.06% (68.87%–71.28%)	78.11% (74.23%–81.23%)	78.74% (76.13%–81.35%)
Random Forest	0.763 (0.731–0.820)	69.16% (67.11%–71.21%)	74.92% (70.85%–78.99%)	69.38% (66.82%–72.03%)

### Derivation and validation of sepsis-associated ARDS phenotypes

**FIGURE 5:**
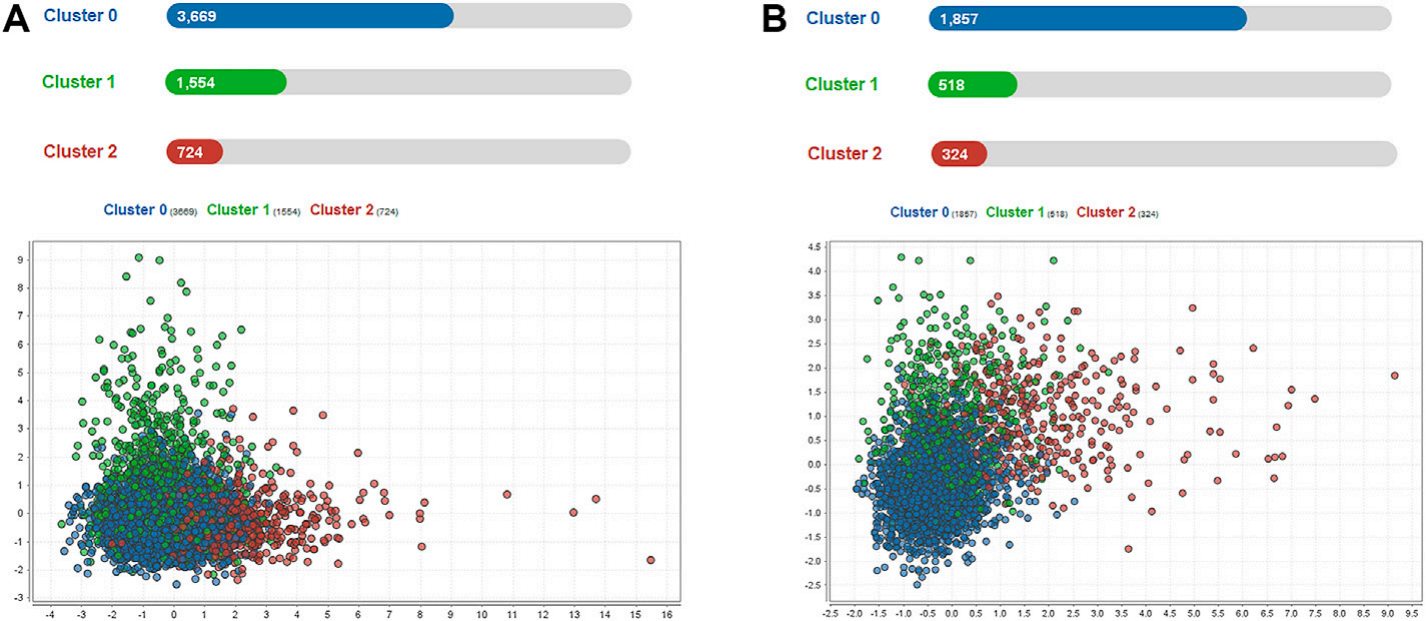
Scatter plot visualization of phenotype assignments by K-means in sepsis-associated ARDS. **(A)**: Scatter plot visualization of phenotype assignments by K-means in the eICU derivation cohort. There were three phenotypes, Clusters 0, 1, and 2, with 3669, 1554, and 724 patients respectively. **(B)**: Scatter plot visualization of phenotype assignments by K-means in the MIMIC-IV validation cohort. There were three phenotypes, Clusters 0, 1, and 2, with 1857, 518, and 324 patients respectively.

**FIGURE 6:**
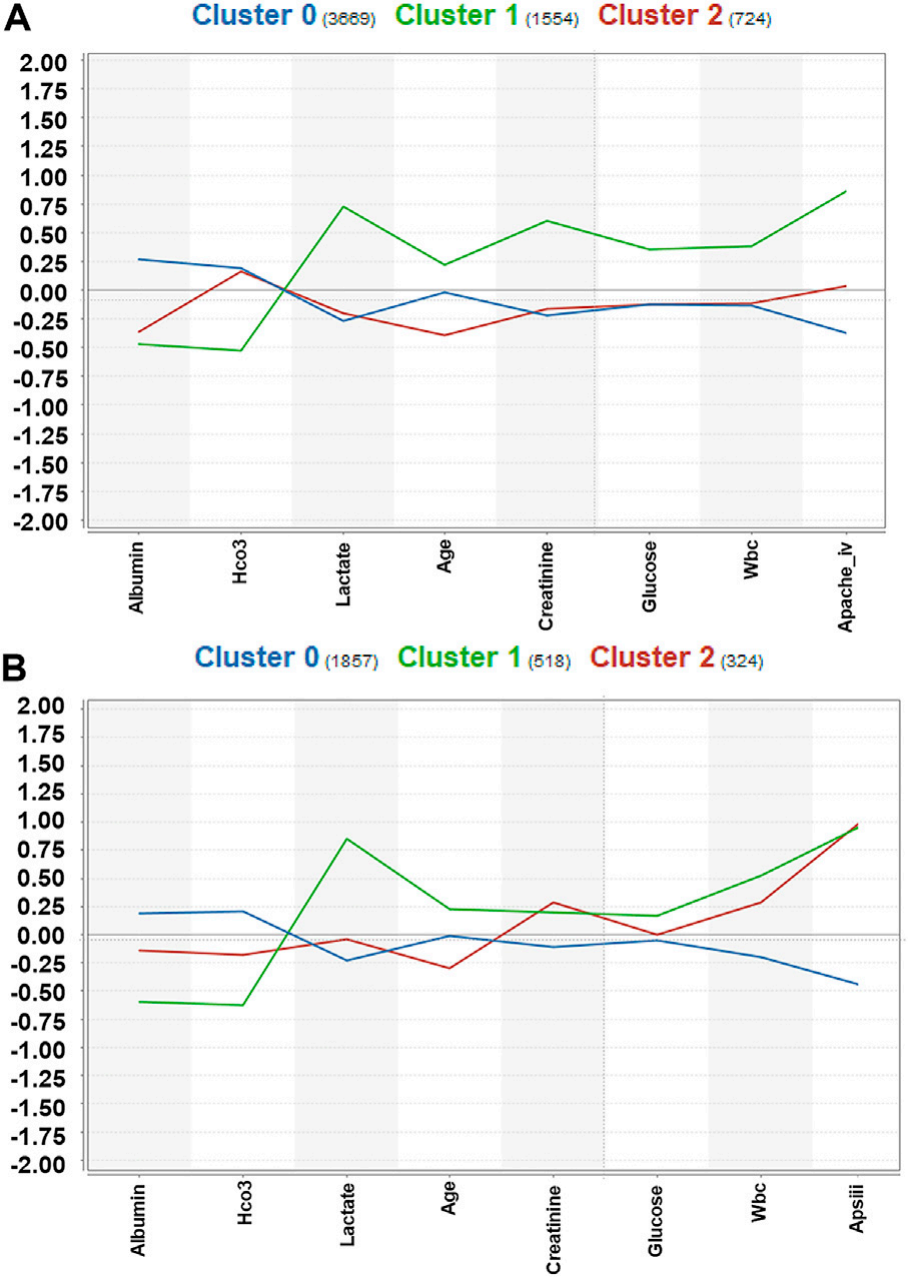
Centroid chart visualization of phenotype assignments by K-means in sepsis-associated ARDS. The laboratory indices were standardized, and the median laboratory indices differed between phenotypes. **(A)**: Centroid chart visualization of phenotype assignments by K-means in the eICU derivation cohort. There were three phenotypes, Clusters 0, 1, and 2, with 3669, 1554, and 724 patients respectively. **(B)**: Centroid chart visualization of phenotype assignments by K-means in the MIMIC-IV validation cohort. There were three phenotypes, Clusters 0, 1, and 2, with 1857, 518, and 324 patients respectively.

A total of 5,947 cases of sepsis-associated ARDS were selected in the eICU database for subgroup clustering by the K-means method, and repeatability was observed in the MIMIC-IV cohort. A total of 2,699 sepsis-associated ARDS patients were selected in the MIMIC-IV database. Regarding the clustering model, after evaluating data availability and the rate of missing clinical variables, we adopt the worst value of each variable selected by the prediction model, which is a total of 8 input variables: APACHE IV, maximum HCO_3_, maximum lactate, maximum creatinine, minimum albumin, maximum glucose, maximum WBC, and age. To evaluate the clustering result, we considered both theoretical and practical factors including: (1) Goodness of fit (2) Adequate large cluster size (3) Salient difference in clinical characteristics between different phenotypes. To determine the optimal number of cluster k, we examined the Gap statistics and the Gap∗ statistics. The optimal number of clustering was determined as 3, which was visualized using a scatter plot (Figure 5) and centroid chart (Figure 6). We also conducted a sensitivity analysis of the clustering strategy. We applied consensus clustering on same data to compare the difference in cluster assignments and phenotype clinical characteristics. We observe a sharp decline in Delta Area of consensus cumulative distribution function from 3 class to 4 class, indicating optimal number of clusters is likely to be 3. Under cluster number = 3, cluster consensus for all cluster was above 0.8 and consensus matrix suggested that goodness of fit was high. The baseline characteristics of the three phenotypes in the eICU derivation cohort are shown in Table 4. The three phenotypes have different clinical characteristics, as follows. Patients in Cluster 0 have lower WBC (median:15.000 K/mcL), lower blood glucose (median:158.000 mg/dl), lower creatinine (median:1.200 mg/dl), lower lactic acid (median:3.000 mmol/L), *p* < 0.001. The age of patients in Cluster 0 was between Cluster1 and Cluster2, and the proportion of comorbidities was also between Cluster1 and Cluster2. These patients were mainly non-traumatic infections and mainly from MICU. Patients in Cluster 1 have the highest WBC (median:18.300 K/mcL), highest blood glucose (median:188.000 mg/dl), highest creatinine (median:2.300 mg/dl), highest lactic acid (median:3.900 mmol/L), *p* < 0.001. Cluster 1 also had the oldest age, highest proportion of comorbidities, mainly non-traumatic infections, and mainly from MICU. Lastly, patients in Cluster 2 have the lowest age, with the albumin, lactic acid, blood glucose and WBC between Clusters 0 and 1. Cluster 2 had the lowest proportion of comorbidities, and was dominated by traumatic infections, which were mainly derived from SICU. We evaluated the repeatability of phenotypes in MIMIC-IV and found that the three phenotypes were similar to those in the eICU derivation cohort (Table 5).

**TABLE 4:** Clinical characteristics and outcomes of three phenotypes in the eICU derivation cohort.

	Cluster0 (*n* = 3,669)	Cluster1 (*n* = 1,554)	Cluster2 (*n* = 724)	*p*
Demographics				
Age, year	67.000 (56.0.78.0)	71.000 (60.0.82.0)	61.000 (50.3.72.0)	<0.001∗∗
Male, n (%)	1856 (50.6%)	909 (58.5%)	385 (53.2%)	<0.001∗∗
Comorbidities				
Hypertension, n (%)	2011 (54.8%)	886 (57.0%)	191 (26.8%)	<0.001∗∗
Diabetes, n (%)	478 (13.0%)	363 (23.3%)	63 (8.7%)	<0.001∗∗
CHF, n (%)	571 (15.6%)	347 (22.3%)	99 (13.6%)	<0.001∗∗
COPD, n (%)	716 (19.5%)	206 (13.3%)	71 (9.8%)	<0.001∗∗
Admission reason				
Traumatic infection, n (%)	741 (20.2%)	349 (22.5%)	497 (68.7%)	<0.001∗∗
Non-traumatic infection, n (%)	2,928 (79.8%)	1,205 (77.5%)	227 (31.3%)	<0.001∗∗
Type of ICU				
MICU, n (%)	2,598 (70.8%)	1,204 (77.5%)	76 (10.5%)	<0.001∗∗
SICU, n (%)	139 (3.8%)	50 (3.2%)	514 (70.9%)	<0.001∗∗
Med-Surg ICU, n (%)	932 (25.4%)	300 (19.3%)	134 (18.6%)	<0.001∗∗
Vitals				
Minimum albumin, g/dL	2.600 (2.5.3.1)	2.300 (1.9.2.6)	2.400 (1.9.2.6)	1
Maximum HCO_3_, mmol/L	23.300 (23.0.27.0)	21.000 (17.0.23.3)	23.300 (22.0.28.0)	1
Maximum lactate, mmol/L	3.000 (1.6.3.4)	3.900 (3.0.8.1)	3.300 (1.7.3.4)	<0.001∗∗
Maximum creatinine, mg/dL	1.200 (0.9.1.9)	2.300 (1.6.3.7)	1.200 (0.9.2.2)	<0.001∗∗
Maximum glucose, mg/dL	158.000 (125.0.205.0)	188.000 (137.0.265.0)	160.000 (124.0.208.0)	<0.001∗∗
Maximum WBC,K/mcL	15.000 (10.5.19.6)	18.300 (12.7.26.8)	15.200 (10.0.20.7)	<0.001∗∗
APACHE IV	68.000 (50.0.75.0)	99.000 (78.0.122.0)	72.000 (65.0.92.0)	<0.001∗∗
Outcome				
LOS, days	2.900 (1.6.5.1)	2.900 (1.1.5.8)	15.900 (12.8.22.1)	<0.001∗∗
Mortality, n (%)	239 (6.51%)	1,170 (75.29%)	215 (29.70%)	<0.001∗∗

^a^ *p* < 0.05 ∗∗*p* < 0.01.

If the variable is a continuous value, it is expressed as the median (interquartile range), and if the variable is a categorical value, it is expressed as a number (percentage of the total). *p* values represent the results of any two comparisons between the three clusters.

HCO_3_: bicarbonate; WBC: white blood count; APACHE IV: acute physiology and chronic health evaluation iv; LOS: length of stay; CHF: congestive heart failure; COPD: chronic obstructive pulmonary disease; MICU: medical ICUs; SICU: surgical ICUs; Med-Surg ICU: medical-surgical ICUs.

**TABLE 5:** Clinical characteristics and outcomes of three phenotypes in the MIMIC-IV derivation cohort.

	Cluster0 (*n* = 1857)	Cluster1 (*n* = 518)	Cluster2 (*n* = 324)	*p*
Demographics				
Age, year	63.000 (53.0.74.0)	67.000 (57.0.78.3)	59.000 (49.3.70.0)	<0.001∗∗
Male, n (%)	970 (52.2%)	320 (61.7%)	170 (52.4%)	<0.001∗∗
Comorbidities				
Hypertension, n (%)	971 (52.2%)	351 (67.8%)	86 (26.5%)	<0.001∗∗
Diabetes, n (%)	559 (30.1%)	179 (34.5%)	48 (14.8%)	<0.001∗∗
CHF, n (%)	340 (18.3%)	125 (24.2%)	56 (17.2%)	<0.001∗∗
COPD, n (%)	405 (21.8%)	123 (23.9%)	57 (17.6%)	<0.001∗∗
Admission reason				
Traumatic infection, n (%)	459 (24.7%)	98 (18.9%)	195 (60.2%)	<0.001∗∗
Non-traumatic infection, n (%)	1,398 (75.3%)	420 (81.1%)	129 (39.8%)	<0.001∗∗
Type of ICU				
MICU, n (%)	1,404 (75.6%)	378 (73.1%)	41 (12.6%)	<0.001∗∗
SICU, n (%)	165 (8.9%)	52 (10.0%)	220 (68.1%)	<0.001∗∗
Med-Surg ICU, n (%)	288 (15.5%)	88 (16.9%)	63 (19.3%)	<0.001∗∗
Vitals				
Minimum albumin, g/dL	2.900 (2.9.3.1)	2.900 (2.2.2.9)	2.900 (2.7.2.9)	1
Maximum HCO_3_, mmol/L	25.000 (22.0.28.0)	21.000 (18.0.25.0)	23.000 (20.0.27.0)	<0.001∗∗
Maximum lactate, mmol/L	3.200 (1.5.3.2)	3.650 (2.5.7.3)	2.650 (1.7.3.7)	<0.001∗∗
Maximum creatinine, mg/dL	1.200 (0.8.2.1)	2.100 (1.3.3.2)	1.800 (1.0.3.6)	<0.001∗∗
Maximum glucose, mg/dL	145.000 (115.0.198.5)	171.000 (126.0.234.0)	156.500 (129.0.205.8)	<0.001∗∗
Maximum WBC,K/mcL	11.700 (8.1.16.3)	17.400 (10.8.25.9)	15.150 (9.5.22.6)	<0.001∗∗
APSIII	48.000 (38.0.61.0)	87.000 (68.0.109.0)	88.000 (73.0.107.0)	<0.001∗∗
Outcome				
LOS, days	2.200 (1.3.4.1)	3.750 (1.7.7.7)	17.000 (13.1.24.1)	<0.001∗∗
Mortality, n (%)	64 (3.45%)	468 (90.35%)	63 (19.44%)	<0.001∗∗

^a^ *p* < 0.05 ∗∗*p* < 0.01.

If the variable is a continuous value, it is expressed as the median (interquartile range), and if the variable is a categorical value, it is expressed as a number (percentage of the total). *p* values represent the results of any two comparisons between the three clusters.

HCO_3_: bicarbonate; WBC: white blood count; APSIII: acute physiology score iii; LOS: length of stay; CHF: congestive heart failure; COPD: chronic obstructive pulmonary disease; MICU:medical ICUs; SICU: surgical ICUs; Med-Surg ICU: medical-surgical ICUs.

### Relationship between phenotype and clinical outcome

The three phenotypes had different clinical outcomes in eICU cohort, as well as in the MIMIC-IV external validation cohort. Cluster 0 had the lowest in-hospital mortality rate (6.51% in eICU and 3.45% in MIMIC-IV), whereas Cluster1 had the highest in-hospital mortality rate (75.29% in eICU and 90.35% in MIMIC-IV). Clusters 0 and 1 had similar median ICU stay length (Cluster0: eICU 2.90 days and MIMIC-IV 2.90 days; Cluster 1: eICU 2.20 days and MIMIC-IV 3.75 days). Meanwhile, Cluster 2 had a moderate in-hospital mortality rate (29.7% in eICU, 19.44% in MIMIC-IV), and the longest ICU stay (15.9 days in eICU, 17 days in MIMIC-IV). Between the two databases, there were significant differences in in-hospital mortality and ICU stay days among the three phenotypes (*p* < 0.05) (Tables 4, 5).

### Therapeutic effects of different PEEP levels in different phenotypes

**FIGURE 7:**
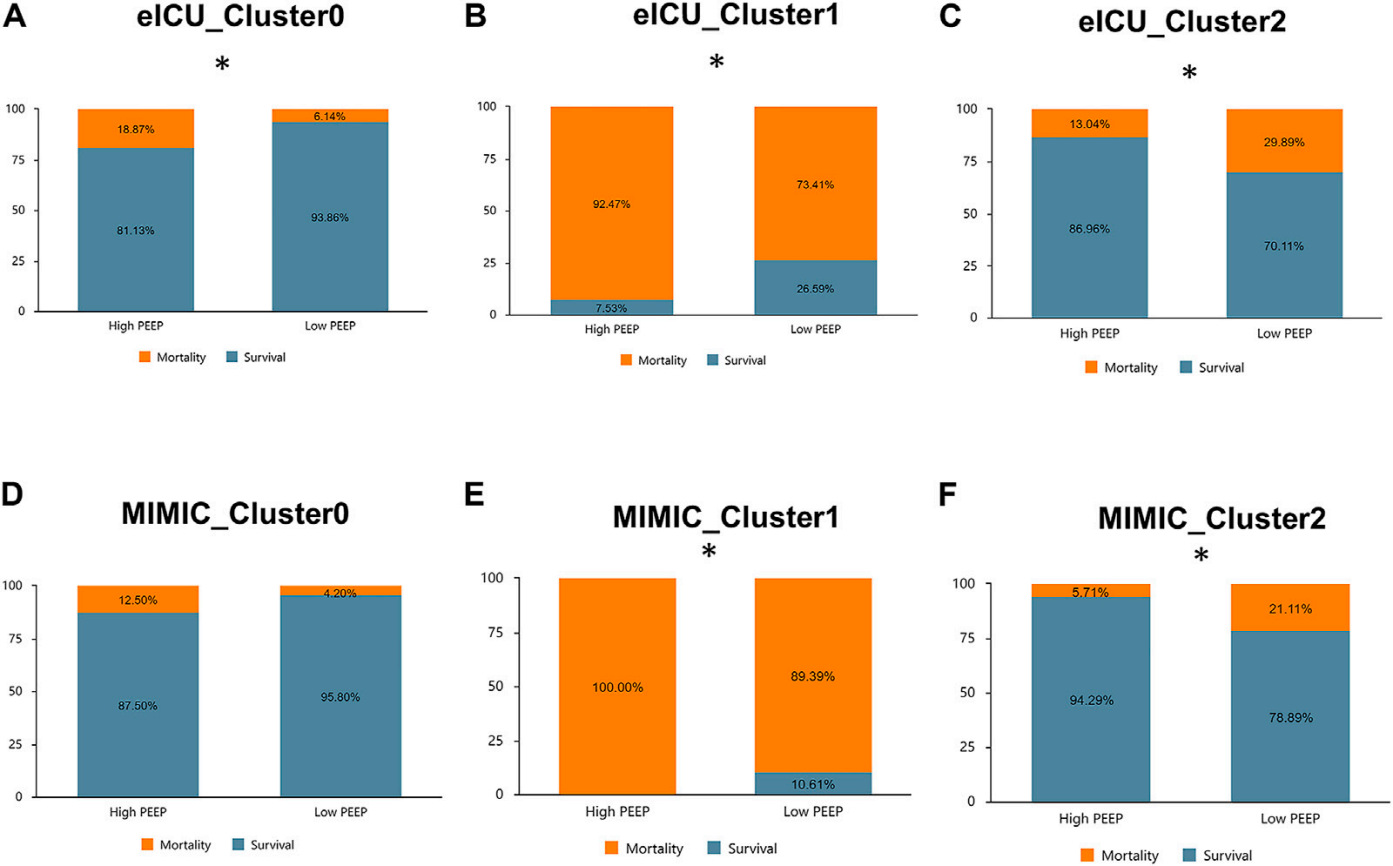
Comparison of in-hospital mortality across phenotypes at high *versus* low PEEP levels in the eICU derived cohort and MIMIC-IV validated cohort. **(A)**: Cluster 0 (eICU): high PEEP had a higher mortality rate than low PEEP (18.87% vs. 6.14%, *p* < 0.05). **(B)**: Cluster 1 (eICU): high PEEP had a higher mortality rate than low PEEP (92.47% vs. 73.41%, *p* < 0.05). **(C)**: Cluster 2 (eICU) : high PEEP had a lower mortality rate than low PEEP (13.04% vs. 29.89%, *p* < 0.05). **(D)**: Cluster 0 (MIMIC-IV): high PEEP had a higher mortality rate than low PEEP (12.50% vs. 4.20%, *p* > 0.05). **(E)**: Cluster 1 (MIMIC-IV): high PEEP had a higher mortality rate than low PEEP (100.00% vs. 89.39%, *p* < 0.05). **(F)**: Cluster 2(MIMIC-IV): high PEEP had a lower mortality rate than low PEEP (5.71% vs. 21.22%, *p* < 0.05).

We divided PEEP within 24 h after admission into high and low PEEP levels, wherein PEEP of >10 cm H_2_O was defined as a high PEEP level. In order not to bias the results, we excluded patients with PEEP missing in this period. The final eICU included 1,473 patient in Cluster 0, 988 in Cluster 1, and 504 in Cluster 2. Cluster 0 in MIMIC-IV had 723 individuals, Cluster 1 had 395 individuals, and Cluster 2 had 324 individuals. The interaction between phenotype and early PEEP strategy was determined. In the eICU derivation cohort, the in-hospital mortality rates of high PEEP in Clusters 0 and 1 were higher compared to low PEEP (*p* < 0.05), whereas in Cluster 2, high PEEP had lower hospital mortality than low PEEP (*p* < 0.05). Similar results were observed in the MIMIC-IV validation cohort (Figure 7). Notably, in the eICU cohort, the mortality rates of low and high PEEP, respectively, were 6.14% (84) and 18.87% (20) in Cluster 0 (*p* < 0.05), 73.41% (657) and 92.47% (86) in Cluster 1 (*p* < 0.05), and 29.89% (130) and 13.04% (9) in Cluster 2 (*p* < 0.05). On the other hand, MIMIC-IV, the mortality rates of low and high PEEP, respectively, were 4.2% (30) and 12.5% (1) in Cluster 0 (*p* > 0.05), 89.39% (358) and 100% (37) in Cluster 1 (*p* < 0.05), and 21.11% (61) and 5.71% (2) in Cluster 2 (*p* < 0.05).

## Discussion

In this study, we developed and validated five machine learning methods using fourteen clinical variables to predict the risk of sepsis-associated ARDS. The AdaBoost (Decision Tree) model exhibited the best performance. We also used rapid key clinical predictors to conduct clinical subgroup clustering for patients with sepsis-associated ARDS. The three phenotypes had different demographics, laboratory tests and outcomes, and these notable responded differently to early high and low PEEP strategies. These results were validated in an additional cohort.

To the best of our knowledge, machine learning for the prediction and clinical subtype classification of sepsis-associated ARDS has not yet been studied. This is the largest study to confirm the prediction and clinical subgroup classification of sepsis-associated ARDS. In this study, whether in the prediction model or in the subgroup clustering, early conventional and easily accessible clinical data are used to ensure the extensibility and clinical transformation of the model, as well as to assist clinicians in the early judgment of patients’ conditions.

The advantage and difference among the development models analyzed in the study are as follow: Naive Bayes is a relatively simple machine learning model, which originates from classical mathematical theory and has a solid mathematical foundation and stable classification efficiency. It has been shown to perform relatively well in the presence of noise, missing data, and irrelevant features (Bi et al., 2019). Logistic Regression is a generalized linear regression analysis model, which is easy to use and explain, but it is sensitive to the multiple collinearity of independent variables in the model. (Edwards et al., 2021). Gradient Boosted Trees is an enhanced integrated model of decision tree, which is flexible and performs well for all kinds of data types (Friedman, 2002). The decision tree model is a easy-to-use classifier model, and the results are easy to explain and robust. Compared with other models, the preparation of data is unnecessary, the requirements for data attributes are not strict, and can achieve good results for large data sources in a relatively short time (Bi et al., 2019). AdaBoost model is a kind of classifier with high precision, which can be used to construct sub-classifiers. Adaboost algorithm provides a framework and rarely appears overfitting phenomenon. However, compared with other weak classifiers, training is more time-consuming (Freund, 1995). Random Forest is a random bagged integrated model of decision tree, which can deal with high latitude data and performs well. The model has strong generalization ability and fast speed, but it is easy to over-fit in some noisy regression or classification problems (Edwards et al., 2021).

Most of the factors involved in previous studies on the diagnosis of sepsis-associated ARDS were inflammatory factors, lung surface proteins or genes, etc. (Wang et al., 2008; Fremont et al., 2010; Ware et al., 2013; Reilly et al., 2018). In our study, clinical indicators within 24 h after admission were used (i.e., APACHE IV/APSIII, maximum HCO_3_, minimum HCO_3_, maximum lactate, minimum lactate, maximum creatinine, minimum creatinine, maximum albumin, minimum albumin, maximum glucose, minimum glucose, maximum WBC, minimum WBC, and age) combined with the machine learning model to obtain an accurate prediction of sepsis-associated ARDS. Studies have found that elevated serum lactate levels and clinical prediction scores were independently associated with the development of ARDS in severe sepsis (Mikkelsen et al., 2013). These two indicators were not only related to the development of ARDS, but also with the prognosis of ARDS patients. A recent Chinese study explored the independent risk factors of community-acquired pneumonia (CAP) complicated with ARDS (Mo et al., 2022), and used an artificial neural network model to predict ARDS in CAP patients. Some of the important predictors in their study were similar with ours, such as age, albumin, creatinine, and blood glucose, and they pointed out that these indicators may be risk factors for ARDS in patients with CAP. In clinical practice, elderly sepsis patients with severe infection, poor nutrition, abnormal renal function, and poor blood glucose control are prone to complications and increasing the risk of ARDS. WBCs are considered the most important effector cells involved in acute inflammation during the pathogenesis of ARDS, with increased WBC heralding the occurrence of ARDS. Abnormal HCO_3_ represents acid-base imbalance, which is seen alongside electrolyte disturbances in the development of ARDS. In conclusion, we believe that several key predictors in this study combined with machine learning models can provide more accurate predictions for the diagnosis of sepsis-associated ARDS patients. Clinical subgroup clustering of sepsis-associated ARDS patients using these key predictors yielded three phenotypes with different clinical characteristics and outcomes.

In 2014, Calfee et al. (2014) divided ARDS patients into two biological phenotypes: hyperinflammatory and hypoinflammatory types. Repeatability verification was conducted in the SAILS cohort (statins for acutely injured lungs from sepsis) in 2018, with approximately 40% of the patients classified as hyperinflammatory phenotypes (Sinha et al., 2018). The team then conducted a series of related studies (Sinha et al., 2020a; Sinha et al., 2020b; Sinha et al., 2022) to validate other ARDS cohorts, continuously simplify the model, and used clinical data to classify biological phenotypes as much as possible. However, the determination of biological phenotype is inseparable from the definition of plasma biomarkers, and thus this is difficult to determine at bedside. In 2021, Liu et al. (2021) used fast and easily available clinical indicators to classify ARDS patients into three clinical phenotypes. These subtypes were not defined by plasma biomarkers and were thus more convenient for rapid clinical application. Type I in their study is similar to Cluster 0 in our study, which included patients with fewer laboratory abnormalities and lowest hospital mortality. Type II is similar to Cluster 2 in our study, which included patients with a more complex condition and moderate mortality. Type III is similar to Cluster 1 in our study, which is closely related to renal insufficiency and acidosis, and had the highest mortality. However, our study is based on subgroup clustering in sepsis-associated ARDS patients, which is more detailed, has less confounding factors, and can explore different clinical characteristics of patients in relatively similar diseases. Alveolar recruitability varies among patients with different severities of ARDS. In patients with mild ARDS, lung tissue recruitability is low, whereas this is high among patients with moderate to severe ARDS (Gattinoni et al., 2006). In our sepsis-associated ARDS patient classification, Cluster 0 patients were the least ill and had poor lung recruitability, which is equivalent to the hypoinflammatory phenotype classification of ARDS by Calfee et al. (2014) in 2014; mortality is lower with low PEEP. In type I ARDS according to the classification of Liu et al. (2021), low PEEP had a lower 60-day mortality rate than high PEEP, which may be because the use of lower PEEP in these patients is sufficient to maintain alveolar inflation and increase functional residual capacity. On the other hand, the patients of Cluster 1 and Cluster 2 have more severe illness, which is equivalent to hyperinflammatory ARDS patients, with a higher mortality rate than Cluster 0. From our classification, these patients can be further subdivided into Clusters 1 and 2. A study in 2017 (Writing Group for the Alveolar Recruitment for Acute Respiratory Distress Syndrome Trial ART Investigators Laranjeira et al., 2017) also pointed out that the use of high PEEP and lung recruitment in patients with moderate to severe ARDS was associated with improved oxygenation but increased mortality. This may be caused by a misclassification of lung morphology in ARDS patients. Therefore, a more specific classification is necessary for moderate to severe ARDS patients. We further classified patients with moderate to severe ARDS into Clusters 1 and 2. Compared to Cluster 0, the overall condition of Cluster 2 is more severe, with higher lung recruitability and younger age, and it is more beneficial to use high PEEP. Cluster 2 patients are mainly from trauma patients, and the clinical benefit is greater when using higher PEEP. From our clinical experience, patients with ARDS from traumatic sources do clinically prefer to use high PEEP compared with other ARDS patients, because these patients have better lung recruitment ability (Burns et al., 2001; Schreiter et al., 2004). For ARDS patients with high alveolar recruitability, a higher PEEP can be selected, which can not only reduce the shear force formed by the periodic collapse of the alveoli, but also avoid ventilator-associated lung injury caused by excessive transpulmonary pressure (Caironi et al., 2010). Cluster 1 is the most severely ill, with an extremely high mortality rate; these patients may not be able to tolerate high PEEP. At this time, the side effects caused by high PEEP outweigh the benefits. Therefore, from our classification of sepsis-associated ARDS patients, lower PEEP is more favorable in Clusters 0 and 1, whereas higher PEEP is better in Cluster 2. In the next step, we will also consider transforming this research into software and other clinical tools to integrate the identification and clustering of sepsis-associated ARDS patients to assist doctors in diagnosis and treatment.

Our study has some limitations. First, many laboratory parameters were removed before model construction due to missing data in over 50% (e.g., pondus hydrogenii, partial pressure of oxygen, and partial pressure of carbon dioxide). Second, the eICU and MIMIC-IV indices of the two databases cannot be completely consistent, thus the APACHE IV and APSIII were interchanged when establishing prediction models and subgroup clustering. Third, missing data was common for some features in the eICU and MIMIC-IV datasets, and thus we performed multiple imputations before statistical analysis. The missing data could result in some bias in our results. Fourth, machine learning cannot avoid the “black box” problem, and it is still a relatively new concept in the field of medicine. Therefore, it is necessary to pilot a prospective implementation study based on system tools in the intensive care environment.

## Conclusion

The use of machine learning in the early diagnosis and classification of sepsis-associated ARDS with easily accessible clinical indicators may assist clinicians in making early diagnosis of the disease, as well as further specify ARDS as a heterogeneous disease, promote individualized and precise treatment, and facilitate clinical transformation and application.

## Data Availability

Publicly available datasets were analyzed in this study. This data can be found here: https://mimic.mit.edu/, https://eicu-crd.mit.edu/.

## Abbreviations

ARDS, Acute respiratory distress syndrome; eICU, The Telehealth Intensive Care Unit; MIMIC-IV, Medical Information Mart for Intensive Care IV; PEEP, Positive end-expiratory pressure; APACHE IV, Acute physiology and chronic health evaluation IV; APSIII, Acute physiology score III; HCO_3_, Bicarbnonate; PT, Prothrombin time; BUN, Blood urea nitrogen; WBC, White blood cell count; IQR, Interquartile range; AUROC, The area under the receiver operating characteristic curve; BMI, Body mass index; LOS, Length of stay; SAILS, Statins for acutely injured lungs from sepsis; CAP, Community-acquired pneumonia; CHF, Congestive heart failure; COPD, Chronic obstructive pulmonary disease; AKI, Acute Kidney Injury; DIC, Disseminated intravascular coagulation; MICU, Medical ICUs; SICU, Surgical ICUs; Med-Surg ICU, Medical-surgical ICUs.
